# A Comprehensive Neurological Study on the Relationship Between Prolactinoma and Alzheimer’s Disease

**DOI:** 10.1002/alz.095681

**Published:** 2025-01-09

**Authors:** Mohamed Khaled Ali Mohamed Ali Zid, Youssef M. Abd El‐Satar, Arwa Ayman Nagah Ahmed Shahin

**Affiliations:** ^1^ Cairo University Faculty of Medicine, Cairo, Cairo Egypt; ^2^ Faculty of Medicine Cairo Univeristy, Egypt, Cairo, Cairo Egypt

## Abstract

**Background:**

Although most pituitary tumors are benign, functioning pituitary tumors are dangerous as they produce and deteriorate the endocrine hormones. Prolactinomas are one of these tumors that arise from lactotroph cells of the anterior pituitary, and they're about 40% of all pituitary adenomas. They secrete high levels of prolactin, which are normally inhibited by dopamine. So, in cases of excess prolactin secretion in prolactinoma, dopamine levels will be elevated as negative feedback. Also, the dopamine system plays a role in cognitive functions and Alzheimer’s disease (AD).

**Method:**

We searched relevant articles through December 2023 in those electronic databases: PubMed, Embase, Web of Science, PsycArticles, MEDLINE, PsycINFO, and the Cochrane Central Register of Controlled Trials; and additional searches for ongoing trials through ClinicalTrials.gov, World Health Organization International Clinical Trials Registry Platform.

**Result:**

**Prolactinomas are found most frequently, with an estimated incidence rate of 10/100,000 person‐years in women of reproductive age. As prolactinomas are the leading pathological case of increasing levels of prolactin, prolactin at first self‐regulates by increasing dopamine release. But in high prolactin states, central dopaminergic neurons become refractory to prolactin, which won’t increase the dopamine level and quite the opposite the dopamine levels would decrease. Some findings suggest that reduced levels of dopamine are associated with AD. Therefore, higher levels of synaptic dopamine would delay the onset of AD. But lately, it has increased the risk of AD**.

**Conclusion:**

These results may suggest clarifying the relationship between dopamine levels in prolactinoma and the incidence of AD. So patients who are suffering from prolactinomas will have a lower risk factor of getting AD at first. But if they haven’t been treated by dopamine agonists or even surgically removal of the tumor, there will be a higher expectation for the occurrence of AD.

